# Redetermination of kovdorskite, Mg_2_PO_4_(OH)·3H_2_O

**DOI:** 10.1107/S1600536812000256

**Published:** 2012-01-11

**Authors:** Shaunna M. Morrison, Robert T. Downs, Hexiong Yang

**Affiliations:** a Department of Geosciences, University of Arizona, 1040 E. 4th Street, Tucson, Arizona 85721-0077, USA

## Abstract

The crystal structure of kovdorskite, ideally Mg_2_PO_4_(OH)·3H_2_O (dimagnesium phosphate hydroxide trihydrate), was reported previously with isotropic displacement paramaters only and without H-atom positions [Ovchinnikov *et al.* (1980 ▶). *Dokl. Akad. Nauk SSSR.* **255**, 351–354]. In this study, the kovdorskite structure is redetermined based on single-crystal X-ray diffraction data from a sample from the type locality, the Kovdor massif, Kola Peninsula, Russia, with anisotropic displacement parameters for all non-H atoms, with all H-atom located and with higher precision. Moreover, inconsistencies of the previously published structural data with respect to reported and calculated X-ray powder patterns are also discussed. The structure of kovdorskite contains a set of four edge-sharing MgO_6_ octa­hedra inter­connected by PO_4_ tetra­hedra and O—H⋯O hydrogen bonds, forming columns and channels parallel to [001]. The hydrogen-bonding system in kovdorskite is formed through the water mol­ecules, with the OH^−^ ions contributing little, if any, to the system, as indicated by the long H⋯*A* distances (>2.50 Å) to the nearest O atoms. The hydrogen-bond lengths determined from the structure refinement agree well with Raman spectroscopic data.

## Related literature

For background to kovdorskite, see: Kapustin *et al.* (1980 ▶); Ovchinnikov *et al.* (1980 ▶); Ponomareva (1990 ▶); Lake & Craven (2001 ▶). For biomaterials studies of hydrated magnesium phosphates, see: Sutor *et al.* (1974 ▶); Tamimi *et al.* (2011 ▶); Klammert *et al.* (2011 ▶). For applications of hydrated magnesium phosphates in the refractories industry, see: Kingery (1950 ▶, 1952 ▶); Lyon *et al.* (1966 ▶); Sarkar (1990 ▶). For applications of hydrated magnesium phosphate in fertilizers, see: Pelly & Bar-On (1979 ▶). For Raman spectra of related systems, see: Frost *et al.* (2002 ▶, 2011 ▶). For correlations between O—H streching frequencies and O—H⋯O donor–acceptor distances, see: Libowitzky (1999 ▶).

## Experimental

### 

#### Crystal data

Mg_2_PO_4_(OH)·3H_2_O
*M* *_r_* = 214.65
Monoclinic, 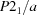

*a* = 10.4785 (1) Å
*b* = 12.9336 (2) Å
*c* = 4.7308 (1) Å
β = 105.054 (1)°
*V* = 619.14 (2) Å^3^
*Z* = 4
Mo *K*α radiation
μ = 0.65 mm^−1^
*T* = 293 K
0.10 × 0.09 × 0.09 mm

#### Data collection

Bruker APEXII CCD area-detector diffractometer
Absorption correction: multi-scan (*SADABS*; Sheldrick, 2005 ▶) *T*_min_ = 0.938, *T*_max_ = 0.944
8463 measured reflections
2231 independent reflections
2008 reflections with *I* > 2σ(*I*)
*R*_int_ = 0.026

#### Refinement

*R*[*F* ^2^ > 2σ(*F* ^2^)] = 0.022
*wR*(*F* ^2^) = 0.056
*S* = 1.07
2231 reflections
129 parameters
All H-atom parameters refined
Δρ_max_ = 0.50 e Å^−3^
Δρ_min_ = −0.34 e Å^−3^

### 

Data collection: *APEX2* (Bruker, 2004 ▶); cell refinement: *SAINT* (Bruker, 2004 ▶); data reduction: *SAINT*; program(s) used to solve structure: *SHELXS97* (Sheldrick, 2008 ▶); program(s) used to refine structure: *SHELXL97* (Sheldrick, 2008 ▶); molecular graphics: *XtalDraw* (Downs & Hall-Wallace, 2003 ▶); software used to prepare material for publication: *publCIF* (Westrip, 2010 ▶).

## Supplementary Material

Crystal structure: contains datablock(s) I, global. DOI: 10.1107/S1600536812000256/wm2577sup1.cif

Structure factors: contains datablock(s) I. DOI: 10.1107/S1600536812000256/wm2577Isup2.hkl

Additional supplementary materials:  crystallographic information; 3D view; checkCIF report

## Figures and Tables

**Table 1:** Hydrogen-bond geometry (Å, °)

*D*—H⋯*A*	*D*—H	H⋯*A*	*D*⋯*A*	*D*—H⋯*A*
O*H*5—H1⋯O*W*6^i^	0.892 (17)	2.497 (18)	3.2408 (10)	141.3 (15)
O*H*5—H1⋯O*H*5^ii^	0.892 (17)	2.511 (18)	3.2033 (14)	134.9 (15)
O*W*6—H2⋯O1^iii^	0.90 (2)	1.77 (2)	2.6518 (10)	165.3 (19)
O*W*6—H3⋯O2^iv^	0.869 (18)	1.849 (19)	2.7097 (10)	170.4 (17)
O*W*7—H4⋯O4	0.88 (2)	1.93 (2)	2.7221 (11)	149.4 (17)
O*W*7—H5⋯O2^iv^	0.83 (3)	2.07 (3)	2.8513 (11)	157 (2)
O*W*8—H6⋯O4	0.83 (2)	1.99 (2)	2.7647 (11)	156.7 (18)
O*W*8—H7⋯O1^i^	0.79 (3)	2.19 (3)	2.9294 (11)	156 (2)

Symmetry codes: (i) 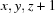; (ii) 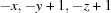; (iii) 
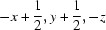
; (iv) 
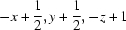
.

**Fig. 1.**
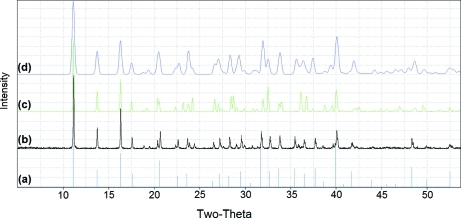
Comparison of the powder X-ray diffraction patterns for kovdorskite. The patterns are shown vertically offset for clarity: (a) by Kapustin et al. (1980), (b) our measurement, (c) calculated pattern based on the data given by Ovchinnikov et al. (1980), and (d) calculated pattern with space group and atomic coordinates reported by Ovchinnikov et al. (1980), but a transformed unit-cell setting (see text).

**Fig. 2.**
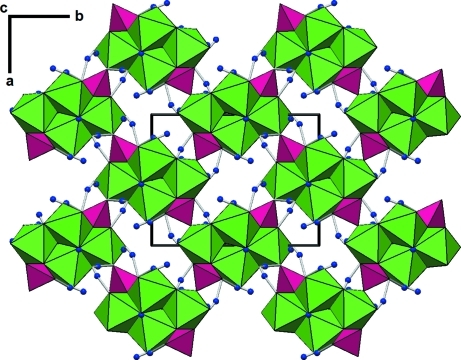
The crystal structure of kovdorskite viewed down c. Green octahedra represent the MgO6 groups and pink tetrahedra the PO4 groups. H atoms are given as blue spheres.

**Fig. 3.**
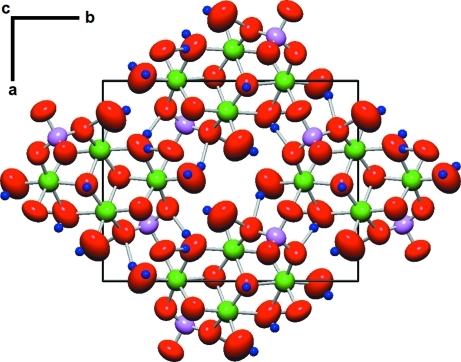
The crystal structure of kovdorskite viewed down c, showing atoms with displacement ellipsoids at the 99% probability level. Green, pink and red ellipsoids represent Mg, P and O atoms, respectively. H atoms are given as blue spheres with an arbitrary radius.

**Fig. 4.**
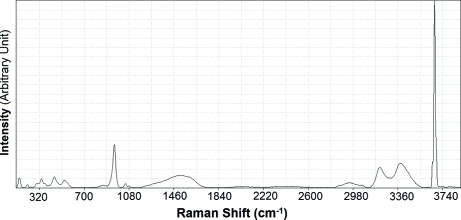
Raman spectrum of kovdorskite.

## supplementary crystallographic information

### Comment

The pseudo-ternary MgO–P_2_O_5_–H_2_O system has been the subject of numerous studies because magnesium phosphates elicit industrial interest. They are used as bonding in refractories (Kingery, 1950; Lyon *et al.*, 1966) and mortars (Kingery, 1952) or as rapid-setting cements (Sarkar, 1990). They also play an important role in the fertilizer industry due to their solubility properties (Pelly & Bar-On, 1979) and in medical research. In particular, newberyite, Mg(HPO_4_).3H_2_O, is a constituent of human urinary stones (Sutor *et al.*, 1974) and has been found to act as a self-setting cement for synthetic bone replacements (Klammert *et al.*, 2011). Moreover, newberyite and cattiite, Mg_3_(PO_4_)_2_.22H_2_O, have shown promising results during in-vivo bone regeneration experiments (Tamimi *et al.*, 2011). In addition to newberyite and cattiite, there are eight other known hydrated Mg-phosphate minerals, including althausite Mg_2_PO_4_(OH), raadeite Mg_7_(PO_4_)_2_(OH)_8_, kovdorskite Mg_2_PO_4_(OH).3H_2_O (or with formula [Mg_2_(OH)(H_2_O)_3_]PO_4_ that better represents the crystal-chemical situation), garyansellite Mg_3_(PO_4_)_2_.3H_2_O, phosphorrösslerite Mg(HPO_4_).7H_2_O, barićite Mg_3_(PO_4_)_2_.8H_2_O, and bobierrite Mg_3_(PO_4_)_2_.8H_2_O.

Kovdorskite from the Kovdor massif, Kola Peninsula, Russia was originally described by Kapustin *et al.* (1980) with monoclinic symmetry in space group *P*2_1_/*c* and unit-cell parameters *a* = 4.74 (2), *b* = 12.90 (4), *c* = 10.35 (4) Å, *β* = 102.0 (5)°. Its structure was subsequently determined by Ovchinnikov *et al.* (1980) based on space group *P*2_1_/*a* and unit-cell parameters *a* = 10.35 (4), *b* = 12.90 (4), *c* = 4.73 (2) Å, *β* = 102.0 (5)°. The resultant *R* factor was 8% with isotropic displacement parameters for all atoms and no locations of H atoms. However, in a meeting abstract, Lake & Craven (2001) reported that kovdorskite crystallizes in space group *P*2_1_/*n* with unit-cell parameters *a* = 4.724, *b* = 12.729, *c* = 10.134 Å, *β* = 102.22°, without presenting other structure information, such as atomic coordinates and displacement parameters. Further chemical and physical analyses on kovdorskite by Ponomareva (1990) revealed that the variation of trace Fe content gives rise to green to blue coloration in this mineral whereas traces of Mn cause a pink coloration.

In the course of identifying minerals for the RRUFF project (http://rruff.info), we noted that the powder X-ray diffraction pattern of kovdorskite we measured on a sample from the type locality displays some obvious inconsistencies with that calculated from the structure model given by Ovchinnikov *et al.* (1980) (Fig. 1). For comparison, plotted in Fig. 1 are also the powder X-ray diffraction data tabulated in the original description of the mineral (Kapustin *et al.*, 1980), which clearly agree with our measured data. In seeking the reason behind the discrepancies between the measured and calculated powder X-ray diffraction data and to better understand the relationships between the hydrogen environments and Raman spectra of hydrous minerals, we re-determined the structure of kovdorskite by means of single-crystal X-ray diffraction.

The crystal structure of kovdorskite is characterized by clusters of four edge-sharing MgO_6_ octahedra that are interconnected by PO_4_ tetrahedra and hydrogen bonds to form columns and channels parallel to [001] (Figs. 2, 3). Within each cluster, there are two special corners where three octahedra are joined. These corners are occupied by hydroxyl ions (OH5). The hydrogen-bonding system in kovdorskite is mainly formed by the H atoms of H_2_O groups, which are all directed toward the channels. The H1 atom (bonded to OH5) contributes little, if any, to the hydrogen bonding system, as indicated by the long H···*A* distances to the nearest OW6 (2.50 Å) or OH5 (2.51 Å).

An examination of our structure data indicates that the discrepancy in the previously published crystallographic data for kovdorskite (Kapustin *et al.*, 1980; Ovchinnikov *et al.*, 1980; Lake & Craven, 2001) and the mismatch between the measured and calculated powder X-ray diffraction pattern result from the inconsistent choice of the unit-cell settings *versus* space groups by Kapustin *et al.* (1980) and Ovchinnikov *et al.* (1980). The space group *P*2_1_/*a* and atomic coordinates given by Ovchinnikov *et al.* (1980) actually correspond to a unit-cell *a* = 10.45, *b* =12.90, *c* = 4.73 Å, and *β* = 104.3°, which can be derived with the transformation matrix (1 0 1 / 0 1 0 / 0 0 1) from their reported cell parameters. The powder X-ray diffraction pattern calculated using this new unit-cell setting, along with reported space group and atomic coordinates, then matches that measured experimentally. In our case, if we choose the unit-cell setting with *a* = 10.3164 (1), *b* =12.9336 (2), *c* = 4.7308 (1) Å, and *β* = 101.231 (1)°, then the corresponding space group is *P*2_1_/*n*. However, if we adopt the setting with *a* = 10.4785 (1), *b* =12.9336 (2), *c* = 4.7308 (1) Å, and *β* = 105.054 (1)°, we have space group *P*2_1_/*a*. The matrix for the transformation from the former setting to the latter one is the same as that given above. In this study, we have adopted the latter unit-cell setting to facilitate a direct comparison of our atomic coordinates with those reported by Ovchinnikov *et al.* (1980).

There have been numerous Raman spectroscopic measurements on a variety of phosphates, including barićite, bobierrite (Frost *et al.*, 2002), and newberyite (Frost *et al.*, 2011). Presented in Figure 4 is the Raman spectrum of kovdorskite. A tentative assignment of major Raman bands for this mineral is made according to previous studies on hydrous Mg-phosphate minerals (*e.g.* Frost *et al.*, 2002, 2011). The most intense, sharp peak at 3681 cm^-1^ is ascribed to the OH5—H1 stretching mode, whereas three relatively broad bands at 3395, 3219, and 2967 cm^-1^ are attributable to the O—H stretching vibrations of the H_2_O molecules, and the very broad bump at 1550 ±100 cm^-1^ to the H_2_O bending vibrations. The O–H···O hydrogen bond lengths inferred from the measured spectrum are in the range 2.62–2.90 Å (Libowitzky, 1999), which compare well with those determined from our X-ray structural analysis (2.65–2.93 Å). Stretching vibrations within the PO_4_ group are responsible for the bands between 840 and 1120 cm^-1^ and bending vibrations for weak bands between 300 and 600 cm^-1^. The bands below 300 cm^-1^ are attributed to lattice vibrational modes and Mg—O interactions.

### Experimental

The kovdorskite specimen used in this study is from the type locality Kovdor Massif, Kola Peninsula, Russia and is in the collection of the RRUFF project (deposition No R050505, http://rruff.info). The chemical composition of the sample was analyzed with a CAMECA SX50 electron microprobe. Only Mg and P, plus very trace amounts of Mn and Ca, were detected. The empirical chemical formula, calculated on the basis of 4.5 O atoms, is Mg_2.00_PO_4.00_(OH).2.67H_2_O, where the amount of H_2_O was estimated by the difference from 100% mass totals.

The Raman spectrum of kovdorskite was collected from a randomly oriented crystal at 100% power on a Thermo Almega microRaman system, using a solid-state laser with a wavenumber of 532 nm, and a thermoelectrically cooled CCD detector. The laser is partially polarized with 4 cm^-1^ resolution and a spot size of 1 µm.

### Refinement

All H atoms were located from difference Fourier syntheses and their positions were refined with isotropic displacement parameters. For simplicity, an ideal chemistry, Mg_2.00_PO_4.00_(OH).3H_2_O, was assumed during the final refinement. The highest residual peak in the difference Fourier maps was located at (0.1815, 0.3311, 0.4211), 0.73 Å from O3, and the deepest hole at (0.7791, 0.7157, 0.4322), 0.50 Å from P1.

### Crystal data

**Table:**

Mg_2_PO_4_(OH)·3H_2_O	*F*(000) = 440
*M**_r_* = 214.65	*D*_x_ = 2.303 Mg m^−^^3^
Monoclinic, *P*2_1_/*a*	Mo *K*α radiation, λ = 0.71073 Å
Hall symbol: -P 2yab	Cell parameters from 4644 reflections
*a* = 10.4785 (1) Å	θ = 2.7–32.5°
*b* = 12.9336 (2) Å	µ = 0.65 mm^−^^1^
*c* = 4.7308 (1) Å	*T* = 293 K
β = 105.054 (1)°	Cuboid, colorless
*V* = 619.14 (2) Å^3^	0.10 × 0.09 × 0.09 mm
*Z* = 4	

### Data collection

**Table:**

Bruker APEXII CCD area-detector diffractometer	2231 independent reflections
Radiation source: fine-focus sealed tube	2008 reflections with *I* > 2σ(*I*)
graphite	*R*_int_ = 0.026
φ and ω scan	θ_max_ = 32.5°, θ_min_ = 3.2°
Absorption correction: multi-scan (*SADABS*; Sheldrick, 2005)	*h* = −15→13
*T*_min_ = 0.938, *T*_max_ = 0.944	*k* = −15→19
8463 measured reflections	*l* = −7→7

### Refinement

**Table:**

Refinement on *F*^2^	Secondary atom site location: difference Fourier map
Least-squares matrix: full	Hydrogen site location: difference Fourier map
*R*[*F*^2^ > 2σ(*F*^2^)] = 0.022	All H-atom parameters refined
*wR*(*F*^2^) = 0.056	*w* = 1/[σ^2^(*F*_o_^2^) + (0.0278*P*)^2^ + 0.150*P*] where *P* = (*F*_o_^2^ + 2*F*_c_^2^)/3
*S* = 1.07	(Δ/σ)_max_ = 0.001
2231 reflections	Δρ_max_ = 0.50 e Å^−^^3^
129 parameters	Δρ_min_ = −0.34 e Å^−^^3^
0 restraints	Extinction correction: *SHELXL97* (Sheldrick, 2008), Fc^*^=kFc[1+0.001xFc^2^λ^3^/sin(2θ)]^-1/4^
Primary atom site location: structure-invariant direct methods	Extinction coefficient: 0.014 (2)

### Special details

**Table:**

Geometry. All e.s.d.'s (except the e.s.d. in the dihedral angle between two l.s. planes) are estimated using the full covariance matrix. The cell e.s.d.'s are taken into account individually in the estimation of e.s.d.'s in distances, angles and torsion angles; correlations between e.s.d.'s in cell parameters are only used when they are defined by crystal symmetry. An approximate (isotropic) treatment of cell e.s.d.'s is used for estimating e.s.d.'s involving l.s. planes.
Refinement. Refinement of *F*^2^ against ALL reflections. The weighted *R*-factor *wR* and goodness of fit *S* are based on *F*^2^, conventional *R*-factors *R* are based on *F*, with *F* set to zero for negative *F*^2^. The threshold expression of *F*^2^ > σ(*F*^2^) is used only for calculating *R*-factors(gt) *etc*. and is not relevant to the choice of reflections for refinement. *R*-factors based on *F*^2^ are statistically about twice as large as those based on *F*, and *R*- factors based on ALL data will be even larger.

### Fractional atomic coordinates and isotropic or equivalent isotropic displacement parameters (Å^2^)

**Table:**

	*x*	*y*	*z*	*U*_iso_*/*U*_eq_	
Mg1	0.15418 (3)	0.48809 (3)	0.04828 (7)	0.00906 (8)	
Mg2	0.49666 (3)	0.21171 (3)	0.93433 (7)	0.00854 (8)	
P1	0.21969 (2)	0.321419 (19)	0.59573 (5)	0.00682 (7)	
O1	0.34918 (7)	0.26654 (6)	0.58522 (15)	0.01002 (14)	
O2	0.13841 (7)	0.24609 (5)	0.73290 (15)	0.01022 (14)	
O3	0.14008 (7)	0.35212 (6)	0.28575 (14)	0.01018 (14)	
O4	0.25621 (7)	0.41919 (6)	0.78609 (15)	0.01050 (14)	
OH5	0.01976 (7)	0.56414 (5)	0.22485 (15)	0.00943 (13)	
OW6	0.16539 (8)	0.64623 (6)	−0.12401 (16)	0.01279 (14)	
OW7	0.32209 (8)	0.53186 (7)	0.35888 (18)	0.01745 (16)	
OW8	0.48594 (8)	0.34594 (7)	1.16317 (18)	0.01686 (16)	
H1	0.0319 (18)	0.5627 (14)	0.419 (4)	0.034 (5)*	
H2	0.160 (2)	0.6770 (16)	−0.298 (5)	0.051 (6)*	
H3	0.2345 (18)	0.6736 (13)	−0.005 (4)	0.030 (4)*	
H4	0.3228 (19)	0.5108 (14)	0.536 (4)	0.035 (5)*	
H5	0.353 (3)	0.591 (2)	0.371 (5)	0.070 (8)*	
H6	0.4269 (19)	0.3840 (15)	1.068 (4)	0.038 (5)*	
H7	0.468 (3)	0.3322 (18)	1.312 (6)	0.063 (7)*	

### Atomic displacement parameters (Å^2^)

**Table:**

	*U*^11^	*U*^22^	*U*^33^	*U*^12^	*U*^13^	*U*^23^
Mg1	0.00832 (15)	0.00862 (16)	0.00998 (15)	0.00010 (12)	0.00191 (11)	−0.00105 (11)
Mg2	0.00828 (15)	0.00792 (16)	0.00926 (15)	0.00010 (12)	0.00198 (11)	−0.00018 (11)
P1	0.00668 (11)	0.00727 (12)	0.00646 (11)	0.00097 (8)	0.00160 (7)	0.00004 (7)
O1	0.0086 (3)	0.0122 (3)	0.0093 (3)	0.0028 (3)	0.0025 (2)	−0.0001 (2)
O2	0.0105 (3)	0.0093 (3)	0.0120 (3)	0.0004 (3)	0.0051 (2)	0.0011 (2)
O3	0.0106 (3)	0.0101 (3)	0.0082 (3)	0.0000 (3)	−0.0006 (2)	0.0015 (2)
O4	0.0115 (3)	0.0097 (3)	0.0105 (3)	−0.0007 (3)	0.0033 (2)	−0.0028 (2)
OH5	0.0103 (3)	0.0095 (3)	0.0082 (3)	0.0003 (2)	0.0018 (2)	0.0000 (2)
OW6	0.0143 (3)	0.0130 (3)	0.0106 (3)	−0.0037 (3)	0.0024 (3)	0.0006 (3)
OW7	0.0181 (4)	0.0163 (4)	0.0149 (4)	−0.0043 (3)	−0.0011 (3)	−0.0001 (3)
OW8	0.0172 (4)	0.0150 (4)	0.0162 (4)	0.0034 (3)	0.0004 (3)	−0.0031 (3)

### Geometric parameters (Å, °)

**Table:**

Mg1—O4^i^	2.0407 (8)	Mg2—OW8	2.0644 (9)
Mg1—OH5^ii^	2.0553 (8)	Mg2—O1	2.0720 (7)
Mg1—OW7	2.0573 (9)	Mg2—O3^v^	2.0991 (8)
Mg1—OH5	2.0641 (8)	Mg2—OW6^iv^	2.2798 (8)
Mg1—O3	2.1124 (8)	P1—O3	1.5390 (7)
Mg1—OW6	2.2162 (8)	P1—O4	1.5419 (7)
Mg2—O2^iii^	2.0365 (8)	P1—O1	1.5434 (7)
Mg2—OH5^iv^	2.0427 (8)	P1—O2	1.5436 (7)
			
O4^i^—Mg1—OH5^ii^	89.62 (3)	O2^iii^—Mg2—O1	91.09 (3)
O4^i^—Mg1—OW7	93.92 (3)	OH5^iv^—Mg2—O1	92.98 (3)
OH5^ii^—Mg1—OW7	173.58 (4)	OW8—Mg2—O1	89.96 (3)
O4^i^—Mg1—OH5	167.00 (3)	O2^iii^—Mg2—O3^v^	90.97 (3)
OH5^ii^—Mg1—OH5	79.87 (3)	OH5^iv^—Mg2—O3^v^	84.20 (3)
OW7—Mg1—OH5	97.28 (3)	OW8—Mg2—O3^v^	92.33 (3)
O4^i^—Mg1—O3	94.58 (3)	O1—Mg2—O3^v^	176.63 (3)
OH5^ii^—Mg1—O3	83.55 (3)	O2^iii^—Mg2—OW6^iv^	172.77 (3)
OW7—Mg1—O3	90.82 (3)	OH5^iv^—Mg2—OW6^iv^	78.34 (3)
OH5—Mg1—O3	91.84 (3)	OW8—Mg2—OW6^iv^	87.62 (3)
O4^i^—Mg1—OW6	95.35 (3)	O1—Mg2—OW6^iv^	87.90 (3)
OH5^ii^—Mg1—OW6	101.25 (3)	O3^v^—Mg2—OW6^iv^	89.74 (3)
OW7—Mg1—OW6	83.77 (3)	O3—P1—O4	109.62 (4)
OH5—Mg1—OW6	79.40 (3)	O3—P1—O1	110.58 (4)
O3—Mg1—OW6	168.99 (3)	O4—P1—O1	108.00 (4)
O2^iii^—Mg2—OH5^iv^	94.57 (3)	O3—P1—O2	109.98 (4)
O2^iii^—Mg2—OW8	99.54 (3)	O4—P1—O2	110.63 (4)
OH5^iv^—Mg2—OW8	165.53 (4)	O1—P1—O2	107.99 (4)

Symmetry codes: (i) *x*, *y*, *z*−1; (ii) −*x*, −*y*+1, −*z*; (iii) *x*+1/2, −*y*+1/2, *z*; (iv) −*x*+1/2, *y*−1/2, −*z*+1; (v) *x*+1/2, −*y*+1/2, *z*+1.

### Hydrogen-bond geometry (Å, °)

**Table:**

*D*—H···*A*	*D*—H	H···*A*	*D*···*A*	*D*—H···*A*
OH5—H1···OW6^vi^	0.892 (17)	2.497 (18)	3.2408 (10)	141.3 (15)
OH5—H1···OH5^vii^	0.892 (17)	2.511 (18)	3.2033 (14)	134.9 (15)
OW6—H2···O1^viii^	0.90 (2)	1.77 (2)	2.6518 (10)	165.3 (19)
OW6—H3···O2^ix^	0.869 (18)	1.849 (19)	2.7097 (10)	170.4 (17)
OW7—H4···O4	0.88 (2)	1.93 (2)	2.7221 (11)	149.4 (17)
OW7—H5···O2^ix^	0.83 (3)	2.07 (3)	2.8513 (11)	157 (2)
OW8—H6···O4	0.83 (2)	1.99 (2)	2.7647 (11)	156.7 (18)
OW8—H7···O1^vi^	0.79 (3)	2.19 (3)	2.9294 (11)	156 (2)

Symmetry codes: (vi) *x*, *y*, *z*+1; (vii) −*x*, −*y*+1, −*z*+1; (viii) −*x*+1/2, *y*+1/2, −*z*; (ix) −*x*+1/2, *y*+1/2, −*z*+1.
